# Quantitative Modeling Assesses the Contribution of Bond Strengthening, Rebinding and Force Sharing to the Avidity of Biomolecule Interactions

**DOI:** 10.1371/journal.pone.0044070

**Published:** 2012-09-14

**Authors:** Valentina Lo Schiavo, Philippe Robert, Laurent Limozin, Pierre Bongrand

**Affiliations:** 1 Aix-Marseille Université, LAI, Marseille, France; 2 Inserm UMR 1067, LAI, Marseille France; 3 CNRS UMR 7333, LAI, Marseille, France; 4 Assistance Publique - Hôpitaux de Marseille (APHM), Hôpital de la Conception, Marseille, France; US Naval Reseach Laboratory, United States of America

## Abstract

Cell adhesion is mediated by numerous membrane receptors. It is desirable to derive the outcome of a cell-surface encounter from the molecular properties of interacting receptors and ligands. However, conventional parameters such as affinity or kinetic constants are often insufficient to account for receptor efficiency. Avidity is a qualitative concept frequently used to describe biomolecule interactions: this includes incompletely defined properties such as the capacity to form multivalent attachments. The aim of this study is to produce a working description of monovalent attachments formed by a model system, then to measure and interpret the behavior of divalent attachments under force. We investigated attachments between antibody-coated microspheres and surfaces coated with sparse monomeric or dimeric ligands. When bonds were subjected to a pulling force, they exhibited both a force-dependent dissociation consistent with Bell’s empirical formula and a force- and time-dependent strengthening well described by a single parameter. Divalent attachments were stronger and less dependent on forces than monovalent ones. The proportion of divalent attachments resisting a force of 30 piconewtons for at least 5 s was 3.7 fold higher than that of monovalent attachments. Quantitative modeling showed that this required rebinding, i.e. additional bond formation between surfaces linked by divalent receptors forming only one bond. Further, experimental data were compatible with but did not require stress sharing between bonds within divalent attachments. Thus many ligand-receptor interactions do not behave as single-step reactions in the millisecond to second timescale. Rather, they exhibit progressive stabilization. This explains the high efficiency of multimerized or clustered receptors even when bonds are only subjected to moderate forces. Our approach provides a quantitative way of relating binding avidity to measurable parameters including bond maturation, rebinding and force sharing, provided these parameters have been determined. Also, this provides a quantitative description of the phenomenon of bond strengthening.

## Introduction

Cell-cell or cell-surface interactions are mediated by highly diverse membrane adhesion receptors. Collectively, these receptors impart attachment a high mechanical strength of typically hundreds of nanonewtons [1], [2] due to multivalent binding [3], [4]. However, the critical step of cell adhesion is probably the formation of the first few bonds. These bonds will generate weak contacts resisting only several tens of piconewtons before subsequent strengthening. A remarkable example is the tethering of leukocytes to endothelial cells in flowing blood through transient interactions between selectins and their ligands [5]. Adhesion efficiency is critically dependent on the kinetics of bond formation and rupture between interacting surfaces in presence of forces.

During the last two decades, remarkable progress was achieved in measuring interactions between surface-attached biomolecules in presence of forces at the single bond level. Investigators used laminar flow chambers, atomic force microscopes or micropipette-based methods (reviewed in 6]. The following conclusions were obtained: i) in the simplest cases [7], [8], the dissociation rate of a ligand-receptor bond exhibited exponential increase in presence of a disruptive force, as suggested by Bell [9]. Bond rupture might be modeled as the passage of a single potential energy barrier in a unidimensional reaction path, following Kramers theory [10]–[13]. ii) In many cases including antigen-antibody [14] streptavidin-biotin [15] or integrin-ligand [16] interaction, bond rupture involved the passage of several sequential energy barriers. These barriers generated multiple bound states for a given ligand-receptor couple. This might provide an explanation for the time-dependent strengthening of antigen-antibody [14], selectin-ligand [17] or streptavidin-biotin [18]–[19] bonds. iii) More recently, two different teams [20]–[21] provided experimental evidence that a disruptive force might paradoxically increase the lifetime of lectin-sugar [20] or P-selectin-PSGL-1 [21] bonds. These force-increasing bonds were dubbed catch-bonds following an early theoretical paper [22]. While the mechanistic basis of the catch-bond phenomenon remains incompletely understood, an important possibility is that bond rupture may not follow an unidimensional path [23] and force might facilitate an alternative rupture path by deforming a multidimensional energy landscape [24]–[26].

A noticeable point is that single bond rupture was studied either by subjecting molecules to a constant force, usually with a flow chamber, or with a steadily increasing force ramp, usually with an atomic force microscope or a biomembrane force probe. In the former case, results were reported as survival curves of bonds subjected to a constant force. In the latter case, authors reported the dependence of rupture force on the rate of force increase, a method called dynamic force spectroscopy [15]. Recently, different authors developed new ways of analyzing data, and they were able to extract the dependence of dissociation rates on instantaneous force from both sets of data [17], [26]–[27]. In some [17], [19] but not all [27] cases, the dissociation rate was found to depend on bond history as well as instantaneous force.

However, while most efforts were focused on single bond studies, much experimental evidence suggests that initial binding is strongly facilitated when at least two bonds can form simultaneously. It has long been reported that the “functional” affinity of divalent IgG or even (Fab’)2 fragments could be 100–1,000 fold higher than that of monovalent Fab fragments [28]–[29]. Further, typical adhesion receptors such as ICAM-1 [30] or PSGL-1 [31] appear as dimers and these dimers are more efficient than monomers in mediating adhesive interactions [30], [31]. This cannot be due to a modification of binding sites, since it was formally shown on ICAM-1 that dimerization was not required to assemble a full binding site [32]. The functional importance of integrin micro- or nano-scale clustering is supported by many experiments [33]–[35] even if conformation is also important [36]. Similar conclusions were found on cadherins [37]. Therefore, it is warranted to explore quantitatively the effects of multivalency on adhesion efficiency.

According to several theoretical studies [38]–[43], the kinetics and mechanics of multivalent attachment rupture should depend on poorly known parameters such as receptor and surface topography, lateral mobility, length and flexibility of membrane anchors, and rebinding rate. Therefore, there is an obvious need for accurate experimental studies of the effect of multivalency on receptor binding properties.

Sulchek et al. [44] used atomic force microscopy to measure the effect of multivalency on attachment mediated by antibodies and MUC-1 antigens connected to surfaces through long polymers: they concluded that forces were shared by parallel bonds. Also, the unstressed dissociation rate was about 40 fold lower with double bonds than with single bonds. Kinoshita et al. [45] used a biomembrane force probe to compare single and double bonds formed by ICAM-1 and LFA-1 receptors borne by polymorphonuclear cells. They concluded that forces were equally shared by divalent bonds. Loritz et al. [46] compared the rupture of single and double antigen-antibody bonds with dynamic force spectroscopy: the yield force of double bonds slightly exceeded that of single bonds.

Here, we used a laminar flow chamber to compare monovalent and divalent attachments between surfaces coated with **low densities** of ICAM-1 monomers or dimers and flowing microspheres coated with a **high density** of anti-ICAM-1 antibodies. The rationale of our approach was as follows: (i) Use monomers to measure the kinetics of single bond rupture in presence of a constant pulling force F of varying intensity. (ii) Use dimers to measure the dissociation rate of attachments mediated by one or two bonds. (iii) Build two algorithms allowing us to determine rupture kinetics of dimer-mediated attachment with two limiting cases: A – When a microsphere is attached by two bonds, then force applies only on one bond. B – When a microsphere is attached by two bonds, force is equally shared between bonds. Each algorithm made use of the experimental rupture kinetics of single bonds (determined with step i) and an adjustable parameter that was the frequency k_r_ of formation of an additional bond between a microsphere attached through one bond and a dimer. This parameter was called rebinding frequency. (iv) Determine with both algorithms A and B the value of parameter k_r_ allowing the best fit between calculated and experimental rupture of dimer-mediated attachments.

As compared with atomic force microscope or biomembrane force probe, the differences are as follows: i) the lag between bond formation and force application was less than 10 milliseconds as compared with typical contact durations of 100 milliseconds with aforementioned techniques. ii) The force applied on a bond remained constant in contrast with the force ramp usually applied with atomic force microscopes. iii) The range of applied forces was narrower with the flow chamber. iv) Since flowing particles sampled a high amount of ligand-coated surfaces, it was possible to use a very low coating density, thus making highly improbable the simultaneous interaction of microspheres with more than one ICAM-1 monomer or dimer. This is a key point for comparing single and double bonds. In another set of experiments, the binding and detachment of nanospheres in absence of flow was quantified. This allowed direct monitoring of force-free bond rupture, instead of merely using extrapolation procedures as usually done with atomic force microscope or biomembrane force probe.

We conclude that bond formation is not an all-or-none process but rather involves progressive strengthening on the subsecond timescale. Strengthening followed a simple empirical law involving a single adjustable parameter. Further, quantitative modeling showed that rebinding of particles maintained by a single bond, i.e. formation of an additional bond by a ligand dimer, was required to account for the force-resistance of attachments mediated by multivalent molecules. Thus, our results provide a quantitative assessment of the importance of multivalent binding in initial attachment. Also, this may provide a quantitative way of accounting for receptor efficiency or avidity.

## Materials and Methods

### Surface and Bead Functionalization

*Glass coverslides* were washed three times with pure ethanol, then rinsed with deionized water and cleaned in piranha solution (H_2_SO_4_/H_2_O_2_ 4:3, Sigma-Aldrich, St Quentin-Fallavier, France) before being coated as previously described [47] with poly-L-lysine (300 kDa, Sigma-Aldrich), then glutaraldehyde and anti-poly-histidine tag IgG1 mAb (AbD Serotec, Oxford, UK). Unreacted aldehyde groups were then blocked with 0.2 M glycine before incubation with 200 µl of 0.04 µg/ml solution of poly-histidine tagged ICAM-1 or Fc(ICAM-1)_2_ chimera (Sinobiological, Beijing, China). The surface density of ICAM-1 groups was estimated at about 1/µm^2^ as obtained after labeling with fluorescent anti-ICAM-1 antibodies and fluorescence determination [47]. The probability that a same anti-histag antibody might bind two poly-histidine-tagged molecules was therefore very low. These estimates were also checked when surfaces were coated with fluorescent nanoparticles and observed with total internal reflection fluorescence (TIRF) microscopy as described below. In this case, glass coverslides were incubated in 200 µl of PBS containing 10 nM of fluorescent streptavidin-coated nanoparticles (605 streptavidin Qdot, Invitrogen, Cergy-Pontoise, France), 10 nM biotinylated anti-ICAM-1 (clone HA58, eBiosciences, San Diego, CA, USA) and 6% BSA.

**Figure 1:**
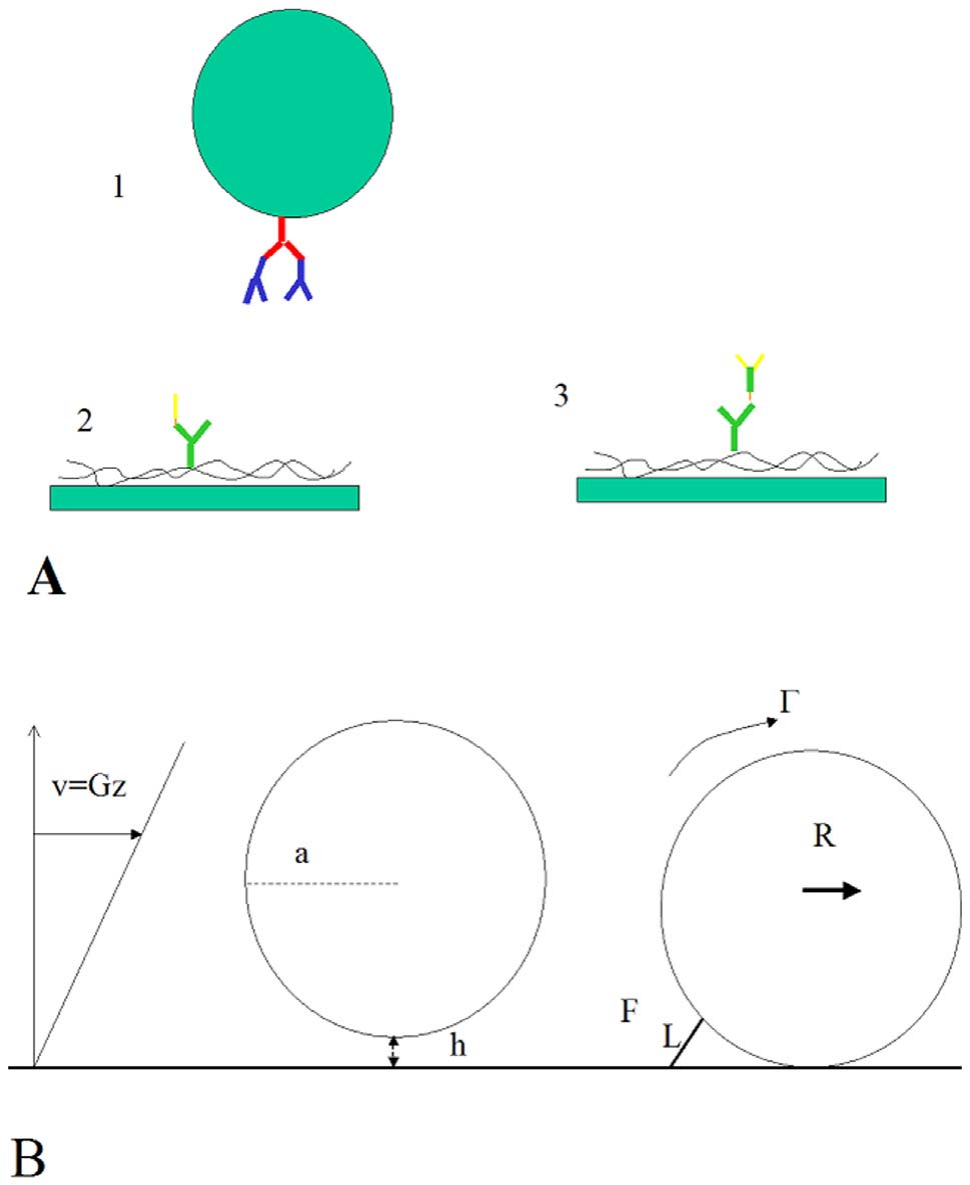
Experimental model. **Fig. 1A**: Microspheres (1) were coated with two immunoglobulin layers made of an anti-immunoglobulin (red) and an anti-ICAM-1 (blue) forming a sequence of four segments of 8 nm length connected by flexible hinges. The surface of flow chambers was coated with polylysine, then an anti-poly-histidine IgG (green) and either a single ICAM-1 moiety terminated with a short poly-histidine (yellow:2) or a Fc(ICAM-1)_2_ fragment (green+yellow: 3). Since the density of tagged ICAM-1 moieties was much lower than that of antibodies, there was a very low probability that an antibody might bind simultaneously two ICAM-1-bearing molecules. **Fig. 1B**: sedimented microspheres of radius a = 2,250 nm were measured to flow with an average distance of about 25 nm to the surface, as a result of brownian motion and short range interactions [45], [50], resulting in a translational velocity (in µm/s) of about 1.215 times the wall shear rate G (in s^−1^). When a molecular bond was formed between the sphere and the surface (right) the force exerted by the flow was dependent on the bond length and was estimated (in piconewton) at about 0.×G [14].

*Microspheres* were tosyl-activated M450 dynabeads of 4.5 µm diameter and 1,500 kg/m^3^ density (Invitrogen) that were coated as previously described [47] first with rat anti-mouse Fc (AbD Serotec, Colmar, France), then with either mouse anti-human ICAM-1 (clone HA58, eBioscience) or mouse IgG1 K isotype control (eBioscience). They were stored at 4°C in a solution of 0.1% BSA and 0.1% sodium azide. For the reader’s convenience, molecular assemblies are depicted on Fig. 1A.

### Microscopy and Data Acquisition for Qdot Binding (Force-free Detachment)

We used TIRF microscopy to measure the surface density of ligands and force-free dissociation kinetics of ICAM-1/anti-ICAM-1 bond. Since the excitation field decreases exponentially from the interface, it penetrates to a depth of only approximately 100–200 nm into the sample. We used an inverted Axiovert 200M microscope (Zeiss, Jena, Germany) equipped with a polarized laser (series 77, LASOS lasertechnik, Jena, Germany), a 100X objective with 1.45 numerical aperture, and a filter cube with 458/10 excitation, 470 dichroic and 605/40 emission filters. Image sequences were recorded with an iXon camera running on iQ software (Andor, Belfast, UK), using an exposure time of 100 ms and a frame rate of 9.6 Hz [48].

Slides were observed immediately after adding streptavidin-coated Qdots and biotinylated anti-ICAM-1, and images were recorded during 20 minutes. Samples were then rinsed five times before resuming observation for about 100 minutes. The Qdot surface density was determined with a multiple-target tracing algorithm [49]. Nonspecific binding was determined on control surfaces that had been treated as described excepted that ICAM-1 addition was omitted. Specific binding was determined by subtracting nonspecific values. Nonspecific binding was always lower than 20% of specific binding. Results were expressed as survival curves by plotting the fraction of Qdots remaining bound versus time after the fivefold wash.

### Data Acquisition in Flow Chamber Experiments

Experiments were performed as previously described [47], [50]. Briefly, microspheres were suspended in PBS supplemented with 1 mg/ml BSA and driven into a parallel-plate flow chamber with an automatic syringe pump (NE500, ProSense BV, Munich, Germany), on the stage of an inverted microscope using a 20X objective and a standard video camera (Sony N50, Clichy, France). The video signal was subjected to real-time digitization (Win TV digitizer, Hauppauge, Paris, France) and compression (DivX codec), then recorded for delayed analysis. Pixel size was 0.5 µm. Particle velocity ranged between about 11 µm/s and 37.5 µm/s. Microsphere tracking was performed with a custom-made software determining the centroid of microsphere images with 40 nm resolution. Full-frame images were disinterlaced allowing 20 ms temporal resolution. The analysis presented in this report is based on the determination of about 27.8×10^6^ microsphere positions, corresponding to a total displacement of 16.5 m and yielding 11,636 binding events.

### Data Analysis

Basic features of motion are depicted on Fig. 1B. It was extensively checked [47], [50] that microsphere motion was consistent with numerical prediction based on low Reynold’s number hydrodynamics [51]. As a result of gravity and short-range colloidal forces, sphere-to-surface distance h fluctuates with a most probable value measured at about 25 nm [52], [53]. As a consequence, the sphere translational velocity parallel to the flow is expected to fluctuate with a peak value u_p_ ≈ 0.54 aG, where a is the microsphere radius and G is the wall shear rate [52]. A sphere was defined as arrested when its displacement δx was lower than 0.5 µm during the following period of time δt = 200 ms. The true arrest duration d_true_ was derived from the apparent arrest duration d_app_ with the correction d_true_ = d_app_ + δt - 2δx/u_p_ [50]. The true number of arrests was estimated by extrapolating at time zero the initial part of experimental survival curve (t≤0.5s) [50]. This segment was nearly linear with a correlation coefficient between time and survival greater than 0.99 (not shown).

Each set of experiments thus yielded the following information: i) the set of arrest durations. Data were used to build survival curves by plotting the fraction s of bonds surviving at time t after formation versus time t. This experimental setup allows direct visualization of the rupture statistics of bonds subjected to a constant force within a range of tens of milliseconds, corresponding to molecule and microsphere repositioning after attachment, to seconds. The statistical uncertainty SD(s) was calculated with binomial law:
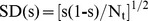
(1)where N_t_ is the total number of arrest and s the fraction of remaining bonds at time t. ii) The **binding frequency f** (per millimeter) was defined as the number of recorded binding events divided by the total trajectory length L of monitored particles. The statistical uncertainty SD(f) was calculated with Poisson’s law as [18]:



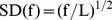
(2)A key advantage of the flow chamber is to yield substantial statistics with surfaces bearing very low densities of receptor molecules. In our experiments, the surface density of ICAM-1 was about 1/µm^2^, yielding a binding probability lower than 10^−3^ per µm bead displacement. This gave a high probability that binding events were generated by single molecular interactions on the basis of Poisson’s law [54]. Another check that was repeatedly performed with this molecular system [55] was that sequential ligand dilutions resulted in proportional decrease of binding frequency without any alteration of survival curves. Thus, we may assume with high confidence that binding events were due to single molecule interactions, which is a key requirement of the present work.

A common difficulty met in studies of rare binding events is the importance of incompletely defined nonspecific binding events. We accounted for this possibility by carefully determining the lifetime distribution and frequency of nonspecific events that were obtained by replacing specific anti-ICAM-1 antibodies by nonspecific immunoglobulins of similar isotype. This information was used to subtract the expected nonspecific contribution from survival curves as was previously done in other studies performed with biomembrane force probe [27].

As shown on Fig. 1B, when a microsphere was maintained at rest by a single bond, the force on the bond could be derived from the standard equations of mechanics, based on the known force F and torque Γ exerted on the sphere by the flow and assuming absence of friction at the sphere-to-surface contact. The tension T on the bond is only weakly dependent on the bond length and is equal to (F+Γ/a) (a/2L)^1/2^ (Fig. 1B and [14] ), yielding T = 0.904 G and T = 0.855 G respectively when surfaces were coated with ICAM-1 or Fc(ICAM-1)_2_ receptors, assuming respectively L = 68 nm and L = 76 nm. Here, T is expressed in piconewton and G in s^−1^.

### Empirical Representation of Survival Curves

It was important to represent experimental data accurately with curves involving a minimal number of parameters. However, a common finding obtained with the flow chamber [14], [18], [47] and atomic force microscopy [17], [19] as well as with soluble phase studies [56]–[57] is that the stability of ligand-receptor bonds is related to their history. An at least partial explanation stems in the multiplicity of binding states and time-dependent passage of ligand-receptor complexes towards the deepest and innermost energy wells. Unfortunately, quantitative account of multiphasic reactions, i.e. reactions involving a number of intermediate states and steps, requires a high number of parameters. Thus, Foote and Milstein [56] needed 8 parameters to describe an antigen-antibody reaction involving only two intermediate states. Here, we looked for a simple way of describing experimental survival curves with only two global parameters. Experimental and fitted curves were compared by calculating the mean squared difference (MSD) between the logarithm of predicted and experimental survival over 19 points spread on the [0,6s] time interval (namely 0 and 1.25^i^/10 - 1, for 1≤ i ≤18). As shown below, an excellent fit was obtained for all tested curves by assuming for the dissociation rate the simple function:
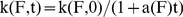
(3)Where F is the force applied on the bond which is assumed to be constant in a given experiment, k(F,t) is the dissociation rate in presence of a disrupting force F and at time t after bond formation, and a(F) is an empirical parameter that is defined as the bond-strengthening rate and is only dependent on F. Writing parameters k(F,0) and a(F) as k and a for short, this yields for the survival curve:



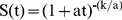
(4)In addition to its simplicity, this formula allows a natural interpretation of k(F,0) as the **initial dissociation rate** and a(F) as the **bond strengthening rate.** It must be emphasized that Eq. 3 should be used for time values on the order of 1/k, i.e. within the second range: It would be meaningless to use it do derive information on the events occurring during the initial ligand-receptor encounter and before force application on the bond, which is on the order of milliseconds.

### Simulation of the Dissociation of Dimer-mediated Attachments

Predicted survival curves were built for divalent ligands by computer simulation. The starting point was an initial attachment with 1 or 2 bonds. The instantaneous dissociation rate k(F,t) was calculated with Eq.3 and parameters k(F,0) and a(F) derived from monomer binding studies. Parameter k(F,t) was used to generate random dissociation events. In some cases a random bond formation with frequency k_r_ was allowed to occur when a single bond existed. Parameter k_r_ may be defined as the rebinding rate since it represents the rate of formation of an additional bond between surfaces already attached with a single bond formed by a divalent receptor. Note that the same parameter was relevant to predict the formation of a bond between a free ICAM-1 and the antibody coated-surface, whether this had already be bound and released, or not. Parameter k_r_ is entirely different from the rate of bond formation between a freely moving sphere and a surface. k_r_ was the only **freely fitted** parameter since k and a were derived from studies made on monomer binding. In addition, two cases were considered, assuming either equal force sharing between two bonds or lack of force sharing. Typically, the time step for a simulation was set at 0.001 second and a theoretical survival curve was built by averaging 5,000 independent time series.

## Results

### Microspheres Displayed Non Specific Binding Events Whose Dissociation Rate Decreased as a Function of Both Time After Arrest and Shear Force

Microspheres coated with anti-ICAM-1 or irrelevant antibodies were driven along surfaces coated with very low densities of monovalent ICAM-1 ligand, on the order of 1 molecule per µm^2^. Microspheres displayed periods of translation with a constant velocity interspersed by arrests. The consequence of using low coating densities was that a significant proportion of binding events were not due to specific ICAM-1/anti-ICAM-1 interactions but rather consisted of so-called nonspecific interactions. This is a common finding in this type of experiments.

**Figure 2:**
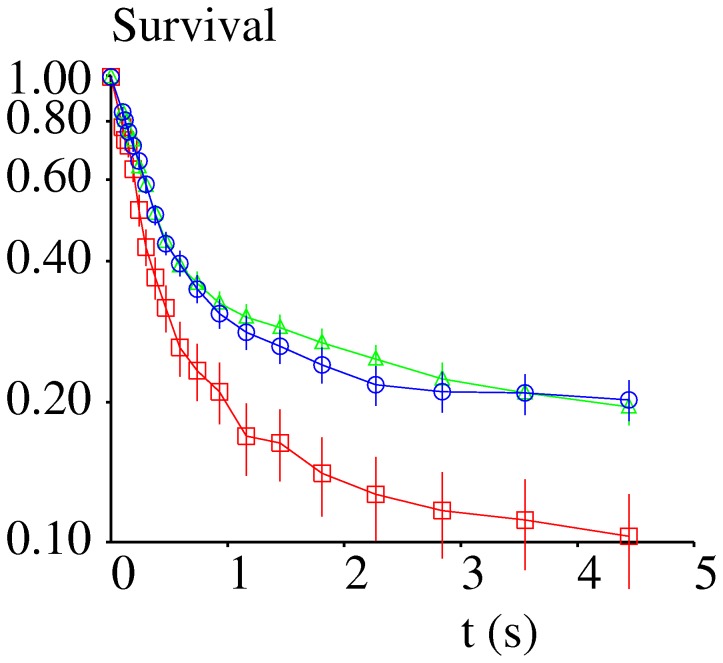
Lifetime of nonspecific arrests. The figure shows the survival curves of binding events recorded between ICAM-1-coated surfaces and microspheres coated with irrelevant antibodies. Squares: wall shear rate 9.3 s^−1^, microsphere velocity 11.25 µm/s, 213 binding events recorded. Crosses: wall shear rate 18.5 s^−1^, microsphere velocity: 22.5 µm/s, 717 binding events recorded. Circles: wall shear rate 29.4 s^−1^, microsphere velocity: 35.7 µm/s, 526 binding events recorded. Vertical bar length is twice the standard error.

The duration of nonspecific binding events was determined by using microspheres coated with isotype-matched immunoglobulin controls instead of anti-ICAM-1 antibodies. As shown on Fig. 2, survival plots of nonspecific arrests displayed a typical time-dependent decrease of dissociation rate. Also, the dissociation rate decreased when the wall shear rate was increased. The average dissociation rate determined during the first 500 ms following bond formation was respectively 2.53 s^−1^, 1.74 s^−1^ and 1.75 s^−1^ when the wall shear rate was 9.3 s^−1^, 19.5 s^−1^ and 30.9 s^−1^. This revealed a clearcut increase of arrest lifetime when the shear rate was increased, as described in other systems [20]–[22].

The frequency of nonspecific binding events was respectively 1.19±0.12 mm^−1^ (9 experiments, 399 arrests), 0.62±0.10 mm^−1^ (32 experiments, 1,362 arrests) and 0.19±0.03 mm^−1^ (21 experiments, 544 arrests) when the wall shear rate was 9.3 s^−1^, 19.5 s^−1^ and 30.9 s^−1^.

### Specific Ligand-receptor Bonds Displayed Lower Dissociation Rate than Nonspecific Bonds, but this Dissociation Rate Increased as a Function of Shear Force

When microspheres were coated with anti-ICAM-1 antibodies instead of nonspecific immunoglobulins, arrest frequency displayed 3.45 fold increase in a total of 67 experiments. Thus, about 71% (i.e. 2.45/3.45) of binding events observed on anti-ICAM-1-coated particles were mediated by specific bonds. It was thus warranted to improve the description of specific events by subtracting the contribution of nonspecific interactions as previously done by other authors [27]. Thus we investigated the variations of the frequency of specific and nonspecific arrests. We tested each ICAM-1-coated slide to determine first the frequency of nonspecific arrests with control microspheres, then the frequency of specific arrests. A strong correlation was found between the frequencies of specific and nonspecific arrests measured on a same slide: indeed, the correlation coefficient r derived from 67 different experiments was 0.8264 (P = 7.3 10^−18^). Secondly, specific binding was fairly low in some experiments, suggesting that coating might alter the conformation of ICAM-1 molecules. Experiments where the ratio between specific and nonspecific bindings was lower than 3 were thus discarded. In remaining experiments, the correlation between specific and nonspecific binding measured on the same slide remained significant. Thirdly, the correlation coefficient between the wall shear rate and the ratio between arrest frequencies measured on control and anti-ICAM-1-coated microspheres was only 0.180 (38 experiments, P = 0.27).

Based on these findings, the fraction P_NS_ of nonspecific binding events was derived from the pooled number of arrests. We obtained P_NS_ = 0.226 (±0.020 SD) on surfaces coated with monomeric ICAM-1 interacting with anti-ICAM-1-coated microspheres and 0.199 (±0.020 SD) on surfaces coated with Fc(ICAM-1)_2_ ligands.

**Figure 3:**
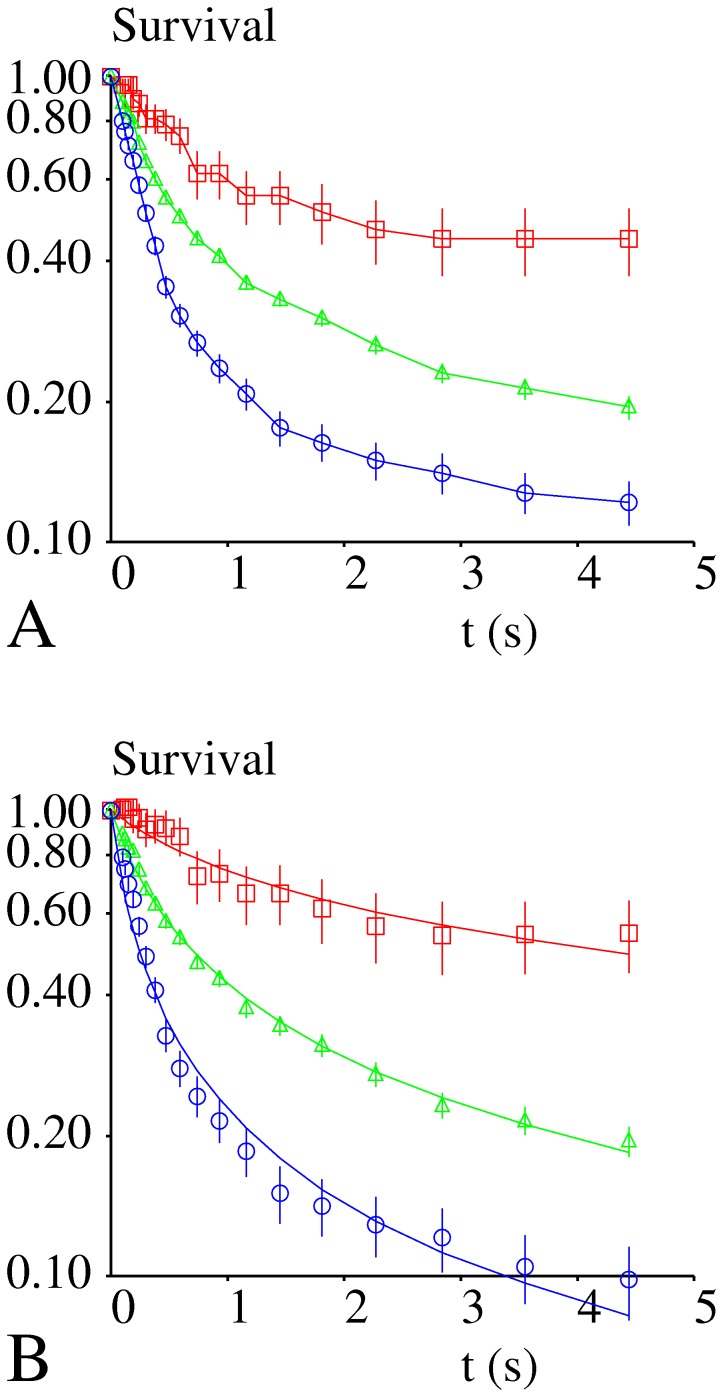
Lifetime of binding events recorded on surfaces coated with monovalent ICAM-1. The figure shows the survival curves of binding events recorded between surfaces coated with low densities of monovalent ICAM-1 and microspheres coated with anti-ICAM-1 antibodies. Red, squares: wall shear rate 9.3 s^−1^, microsphere velocity 11.25 µm/s, 47 binding events recorded. Green, triangles: wall shear rate 18.5 s^−1^, microsphere velocity 22.5 µm/s, 1,725 binding events recorded. Blue, circles: wall shear rate 29.4 s^−1^, microsphere velocity 35.7 µm/s, 936 binding events recorded. **Fig. 3A**: the raw values were used. **Fig. 3B**: values were corrected to account for nonspecific events as explained. The curves represented the best fits of experimental curves with Eq. 2. Squares: Force on bond is 8.37 pN, k(F,0) = 0.441 s^−1^, a(F) = 1.099 s^−1^, red line: calculated fit, MSD = 3.7 10^−3^. Crosses: Force on bond is 16.75 pN, k(F,0) = 1.735 s^−1^, green line: calculated fit, MSD = 0.99 10^−3^. Circles: Force on bond is 26.61 pN, k(F,0) = 4.603 s^−1^, a(F) = 6.149 s^−1^, MSD = 12.4 10^−3^. Vertical bar length is twice standard error.

The survival plots of attachments formed between specific antibodies and surfaces coated with monomeric ICAM-1 are shown before (Fig. 3A) and after (Fig.3B) correcting for non specific arrests. The difference between these plots demonstrated the importance of this correction. Indeed, the average dissociation rate measured during the first 500 ms under the lowest shear rate was respectively estimated at 0.577 s^−1^ and 0.254 s^−1^ before and after correction. In contrast with nonspecific arrests, the lifetime of specific bonds was decreased when the shear rate was increased. Average dissociation rates measured during the first 500 ms were respectively 0.254 s^−1^, 0.532 s^−1^ and 1.059 s^−1^ when the pulling force exerted on bonds was estimated at 8.4, 16.7 and 26.6 pN.

### Divalent Attachment Results in Markedly Increased Resistance to Shearing Forces as Compared to Monovalent Attachment

**Figure 4:**
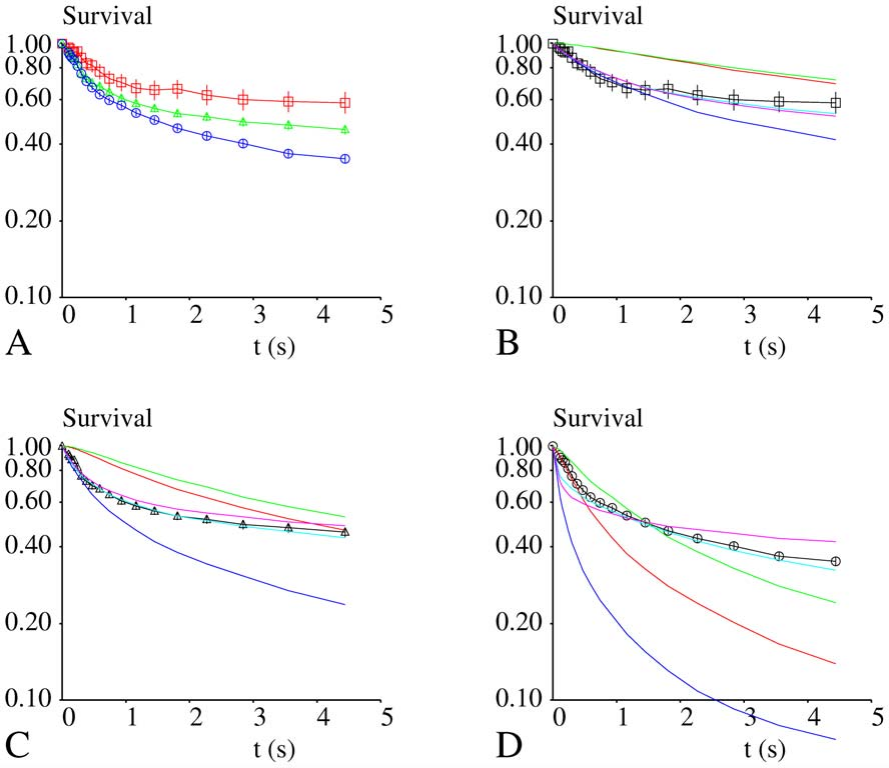
Lifetime of binding events recorded on surfaces coated with divalent ICAM-1. The figure shows the survival curves of binding events recorded between surfaces coated with low densities of Fc(ICAM-1)_2_ molecules and microspheres coated with anti-ICAM-1 antibodies. **Fig. 4A**: all survival curves corrected for non specific arrests. Squares: wall shear rate 10.3 s^−1^, microsphere velocity 12.5 µm/s, 122 binding events recorded. Force on bond is 8.80 pN. Triangles: wall shear rate 18.5 s^−1^, microsphere velocity 22.5 µm/s, 1009 binding events recorded. Force on bond is 15.84 pN. Circles: wall shear rate 30.9 s^−1^, microsphere velocity 37.5 µm/s, 1939 binding events recorded. Force on bonds is 26.40 pN. **Fig. 4B.** Squares: experimental data, lowest wall shear rate: 10.3 s^−1^. Theoretical curves are shown for the following conditions: two bonds at time zero, k_r_ = 0, force not shared (red) or shared (green) between bonds. One bond at time zero, k_r_ = 0, (blue), one bond at time zero, k_r_ = 0.3 s^−1^, force not shared (cyan) or shared (purple) between bonds. **Fig. 4.C**. Triangles: experimental data, intermediate wall shear rate 18.5 s^−1^. Theoretical curves are shown for the following conditons: Two bonds at time zero, k_r_ = 0, force not shared (red) or shared (green) between bonds. One bond at time zero, k_r_ = 0 (blue),. one bond at time zero, k_r_ = 1.1 s^−1^, force not shared (cyan) or shared (purple) between bonds. **Fig. 4D**. Circles: experimental data, highest shear rate 30.9 s^−1^. Theoretical curves are shown for the following conditions: Two bonds at time zero, k_r_ = 0, force not shared (red) or shared (green) between bonds. One bond at time zero, k_r_ = 0 (blue), one bond at time zero, k_r_ = 12 s^−1^, force not shared, (cyan) or k_r_ = 6s^−1^, force shared (purple) between bonds. Vertical bar length is twice the standard error.

Microspheres were made to bind surfaces coated with low densities of divalent Fc(ICAM-1)_2_ ligand, and survival curves are shown on Fig. 4A. Interactions measured under the lowest wall shear rate were fairly comparable to those observed with monomeric ICAM-1, with a survival slightly higher than 50% at time 5 second. However, the sensitivity to shear was much lower since the highest force reduced potentially divalent binding by only 40%, i.e. **1.7 fold decrease**, 5 s after bond formation, whereas the survival of monovalent binding exhibited **6 fold** decrease under the same conditions.

### Even in Absence of Shearing Forces, Divalent Attachment Results in much Higher Lifetime than Monovalent Attachment

**Figure 5:**
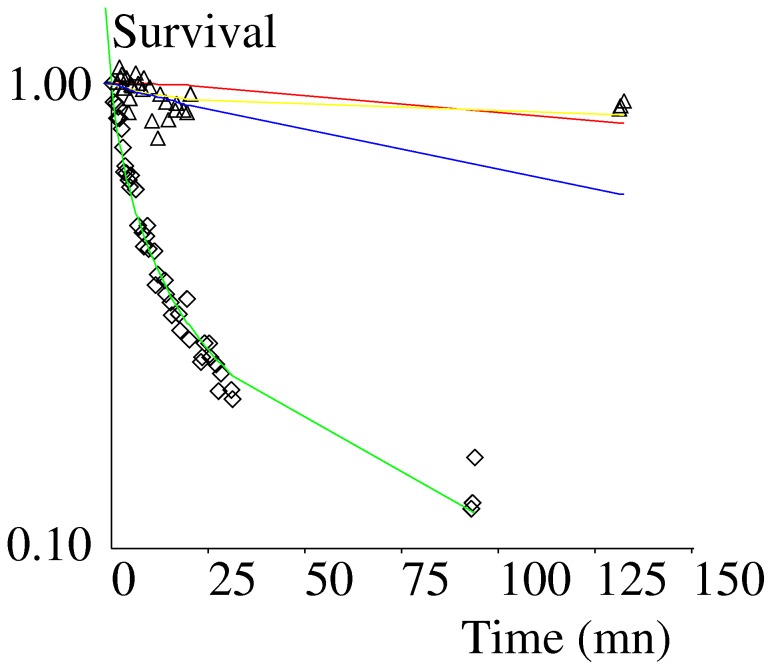
Force free survival of attachments between microspheres and ICAM-1-coated surfaces. Anti-ICAM-1-coated Qdots were incubated with surfaces coated with monovalent (diamonds) or divalent (triangles) ICAM-1 and spontaneous detachment was determined by counting bound Qdots on a microscope area of 1 µm^2^. Each point represents about 800–1000 particles. Green line: fit of monovalent binding with constants k(0,0) = 0.167 mn^−1^ and a(0) = 0.252 mn^−1^ (Eq. 2). Red line: calculated survival curve for dimers, two bonds at time zero, k_on_ = 0, MSD = 0.0105. Blue line: calculated survival for dimers, one bond at time zero, k_on_ = 0, MSD = 0.025. Yellow line: calculated survival curve for dimers, one bond at time zero, k_on_ = 1.4 mn^−1^, MSD = 0.0052.

We monitored the release of Qdots bound to surfaces coated with monomeric or dimeric ICAM-1 ligand through anti-ICAM-1, in absence of flow. Since binding was allowed to occur during a period of 20 minutes, ICAM-1/anti-ICAM-1 bonds were expected to have matured sufficiently to generate more durable attachment than obtained after less than a few seconds of contact. As shown on Fig. 5, attachment was much more durable than observed in the flow chamber, as expected. Further, the difference between monovalent and divalent attachment was still more impressive than found with the flow chamber, since no substantial Qdot release was observed during 120 minutes when binding was potentially divalent, while 90% detachment was observed within 100 minutes when attachment was monovalent.

### Single Bond Rupture under Forces is Well Described by Two Parameters: the Initial Dissociation Rate and the Strengthening Rate

As shown on Figure 3, single-bond attachments displayed time-dependent decrease of dissociation rate. Thus, we used Eq. 3 as a simple way of achieving an empirical description of bond rupture during the timescale of experiments. The basic assumption was that the initial dissociation rate k_0_ was divided by (1+at) at time t, thus introducing a single strengthening parameter a. As shown on Fig. 3B and Table 1, this simple formula allowed a close fit with experimental values, since the mean square of relative difference (MSD) between fitted curves and experimental points was less than 0.0015. Interestingly, this formula also allowed a satisfactory fit of force-free detachment data (Fig. 5 and Table 1).

**Table 1:** Estimated parameters for rupture of ICAM-1/anti-ICAM-1 bonds subjected to force.

Wall shear rate (s^−1^)	Force (pN)	k(F,0) (s^−1^)	a(F) (s^−1^)	k(F/2,0) (s^−1^)	a(F/2) (s^−1^)
0	0	0.168	0.512	0.168	0.512
10.3	8.80	0.519	1.171	0.295	0.775
18.5	15.84	1.277	2.270	0.463	1.078
30.9	26.4	4.934	6.126	0.911	1.772

**Figure 6:**
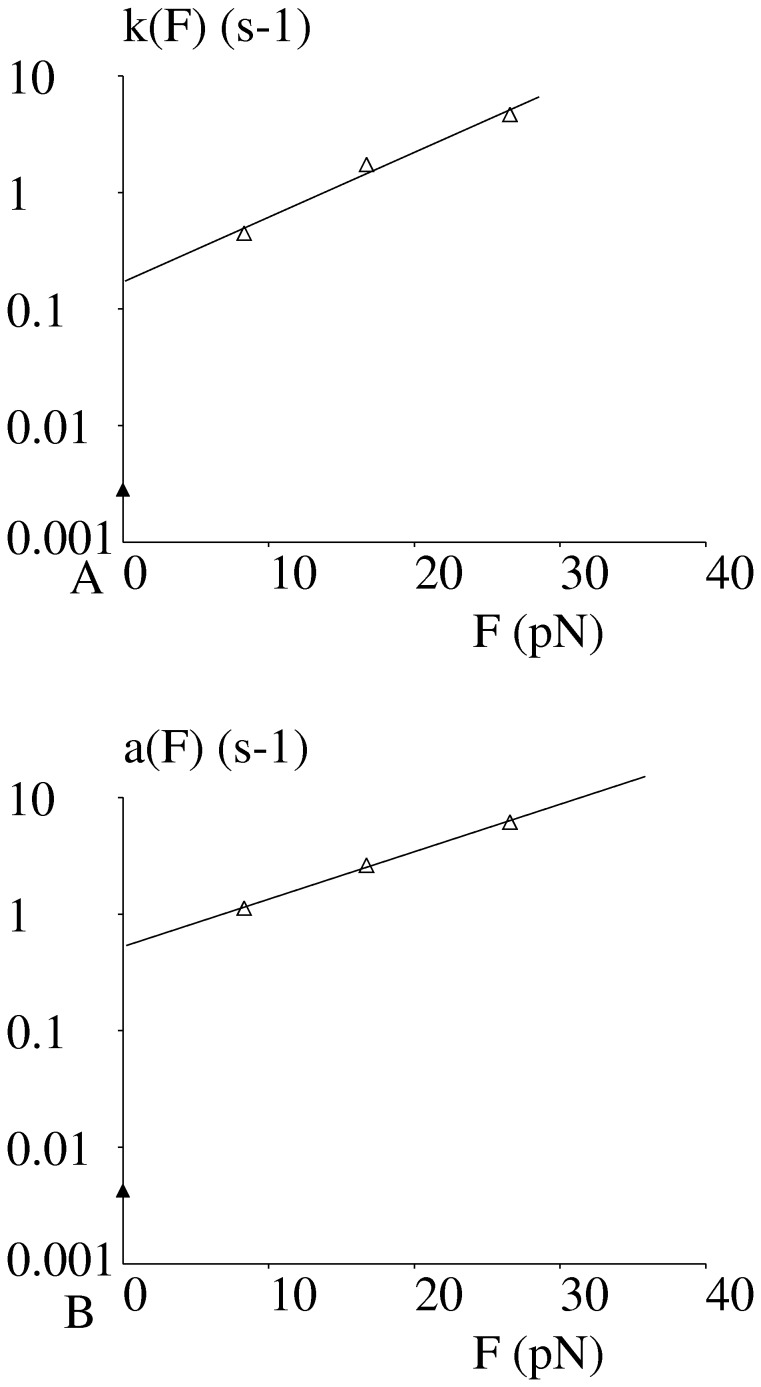
Force dependence of off-rate and bond strengthening parameter. The dependence of bond initial dissociation rate (Fig. 6A) and strengthening rate (Fig. 6B) on applied forces are shown. Open triangles represent data obtained with the flow chamber and Filled triangles represent data obtained with Qdots in absence of flow. Since time scales were markedly different, only results obtained with the flow chamber were used to estimate the rupture behavior of bonds formed with Fc(ICAM-1)_2_ in the flow chamber, with either force sharing or non sharing assumption. Estimated values are shown on Table 1.

The numerical values of parameters used to build simulated survival curves of attachments formed by microspheres and Fc(ICAM-1)_2_ - coated surfaces are shown as derived by extrapolating results displayed on Fig. 6.

Experimental data were used to estimate the dependence of the initial dissociation rate k(F,0) and bond strengthening parameter a(F) on the force F applied to a bond under tension. As shown on Fig. 6, results suggested a linear dependence of k and a on exp(F), similarly to Bell’s law. The regression lines shown on Fig. 6 were used to estimate the dependence of k and a on F in a force interval of about [0, 35 pN]. It must be emphasized that the range of data points was insufficient to yield detailed information on the force dependence of parameters a and k. The numerical values obtained under force-free conditions were not deemed comparable to those estimated under flow extrapolated at zero forces since the time scales of periods between bond formation and rupture measurement were respectively on the order of minutes (Qdots) and seconds (flow chamber).

### The Two-parameter Description of Single Bond Rupture Allows Derivation of Rebinding Rates between Surfaces Exposing Divalent Receptors and Linked by a Single Bond

We used the numerical data summarized on Table 1 to build simulated survival curves in order to test different independent assumptions: i) a force applied on a divalent attachment is applied on a single bond, or it is equally shared between both bonds (**no force sharing or force sharing**). ii) There is a zero or non-zero bond forming rate k_r_ between a microsphere and a surface linked by a single bond involving a divalent receptor (**no rebinding or rebinding**). iii) During the first milliseconds of attachment between anti-ICAM-1 coated microspheres and Fc(ICAM-1)_2_-coated surfaces, a single or two bonds are formed (**monovalent or divalent initial attachment**). A number of simulated curves are displayed on Figures 4B-D and compared to experimental data. The following conclusions could thus be drawn sequentially:

First, we found that the **initial attachment was monovalent**. This was seen most clearly with the lowest velocity (Fig. 4.B): The MSD between experimental and theoretical curves (that were obtained by assuming that two bonds were formed at time zero) was higher than 0.7. Further, since calculated survival was higher than experimental values, the fit would have been still worse if the rebinding parameter k_r_ was nonzero. Also, similar findings were found with and without force sharing. Thus, whatever the other parameters, it could be safely concluded that initial attachment was monovalent.

Secondly, we found that a satisfactory fit between experimental and calculated survival curves required the occurrence of additional bond formation (ie nonzero k_r_ parameter). Our reasoning is illustrated on Fig. 4:

For the lowest shear velocity, in absence of rebinding, the simulated curve was visibly different from experimental one, with a MSD of 0.0135. However, a satisfactory fit could be obtained with k_r_ = 0.3 s^−1^, yielding a MSD of 0.0022 and 0.0017 respectively with force sharing or not sharing (Fig. 4B).
For the intermediate shear velocity, a good fit was obtained with both assumptions of force sharing and not sharing and k_r_ = 1.1 s^−1^. The MSDs were respectively 0.0019 and 0.0008. In absence of rebinding, MSDs were higher than 0.04 with 1 or 2 bonds, whatever the assumption concerning force sharing (Fig. 4.C).
For the highest velocity, a fairly poor fit could be obtained with both force sharing and no force sharing assumptions, MDSs were respectively 0.023 (k_r_ = 6 s^−1^) and 0.009 (kr = 12 s^−1^) in two representative simulations, which is fairly reasonable, but the shape of experimental and predicted curves were clearly different (Fig. 4D).

In conclusion, simulated curves could only be fitted to experimental data by assuming that i) initial attachment was monovalent, ii) rebinding could occur, and iii) force sharing between bonds had a small influence on survival curves which made it difficult to draw definitive conclusions concerning this point.

### Both Bond Strengthening and Rebinding Play a Key Role in Contributing the Divalent Bond Capacity to Resist Moderate Forces

The conclusion of our study is that rebinding, bond strengthening and to a lesser extent force sharing all have the capacity to contribute the divalent attachment resistance to forces. Our model allows some estimate of the relative contribution of these effects, although this is not fully significant since they are not additive: We built survival curves for the highest force (Figure 4D) with the following assumptions: i) force sharing, rebinding and bond strengthening, ii) rebinding and bond strengthening, iii) force sharing and bond strengthening, and iv) force sharing and rebinding. While attachment survival in presence of the highest force is about 44% after 5 seconds with all three mechanisms simultaneously allowed, it would be about 1.9 fold lower in absence of force sharing, which was felt to represent a modest change, 5.9 fold lower in absence of bond formation, and 24.3 fold lower in absence of bond strengthening. These figures provide a quantitative insight into the hierarchical importance of these mechanisms.

## Discussion

During the last fifteen years, much work was done to describe the formation and rupture of bonds between surface-attached biological receptors and ligands at the single molecule level. All these studies revealed a growing complexity of ligand-receptor interaction. It was first considered that the kinetic rates of bond formation and rupture could give a reasonable account of ligand-receptors interactions [58]. It was then recognized that an independent parameter must be added to account for the bond mechanical strength. In many cases this was done with Bell’s empirical formula [9], [59]. Other factors of complexity were that bond formation and rupture behaved as multi-step phenomena with an impressive hierarchy of binding states [14], [15], [27] and other bonds displayed so-called catch-bond behavior, i.e. the bond lifetime was increased by moderate pulling forces [20]–[22]. In comparison, fewer studies were devoted to the theoretical [38]–[42] or experimental [43]–[46] behavior of multivalent attachments.

The strategy followed in this study was to use a model system in order to produce a working description of monovalent attachments, then to measure and interpret the behavior of dimer-mediated attachments under force. The main conclusion are that i) A new empirical parameter called the **bond strengthening** rate is required to account for the maturation of newly formed bonds. While the structural basis of our results remains to be investigated, it must be emphasized that the conclusion that ligand-receptor bonds are expected to display extensive maturation with a timescale ranging from subsecond to hundreds of seconds or more is consistent with the expected complexity of energy landscapes and experimental reports on kinetic rates ranging between tenths of s^−1^ [56] or less [60] and more than 100 s^−1^ [50]. ii) Both bond formation (as accounted for by the rebinding parameter) and bond strengthening play a major role in increasing the survival of divalent attachments as compared to monovalent attachments. The dramatic difference between monomer-mediated and dimer-mediated attachments made with a given receptor-ligand couple may provide an explanation for the common finding that many cell membrane receptors act as dimers.

The present study provides both a detailed example of this general concept and a simple experimental and theoretical framework for data analysis. In order to fully assess the significance of our results, several points need to be discussed.

Firstly, the flow chamber operated under low shear rate is well suited to study the behavior of single bonds subjected to moderate forces [61]. Indeed, when the microspheres we used were subjected to a wall shear rate of 10 s^−1^, they experienced a pulling force of only 1.62 pN, and their velocity was about 12 µm/s. Thus, during a 20 millisecond interval corresponding to the standard acquisition rate, their displacement of 240 nm was easily measurable with our tracking software, allowing optimal sensitivity for detecting the weakest binding events. Also, since microspheres scanned extensive areas, it was possible to use very low coating densities of ligands, thus providing optimal elimination of binding events involving more than one ligand, which was a key requirement in our study.

Secondly, we assumed that the rupture of specific sphere-to-surface attachments resulted from the rupture of transient ICAM-1/anti-ICAM-1 interactions rather than his-tag/anti-hist-tag or Fc/anti-Fc interactions. This assumption was supported by the following two points: first, a general finding with most ligand-receptor couples was that the off-rate exhibited steady decrease during the first tens of seconds or minutes following bond formation. This makes more likely that rupture events were due to the disruption of the newest bond even if it was as strong as the streptavidin-biotin interaction [18]. Secondly, if most ruptures involved his-tag/anti-his-tag or Fc/anti-Fc interaction, no difference would be found between the monovalent and divalent ICAM-1/anti-ICAM-1 attachments, in contrast with our experimental data.

Thirdly, our results illustrate the importance of so-called nonspecific binding events, and the importance of taking care of them as was indeed recognized by other investigators [27]. We provided some quantitative information on these events, and we found that their lifetime was of the same order of magnitude as those generated by single bonds. The difficulty of ruling out artifacts potentially generated by this occurrence is certainly the most demanding part of data collection. This raises at least two specific points. i) It is important to rule out the possibility that the progressive development of nonspecific interactions between surfaces held together by a specific bond might artefactually decrease experimental dissociation rates. This possibility is made unlikely by our recent finding that dissociation rates measured between surface-attached molecules with the flow chamber were consistent with results obtained on soluble ligands with surface plasmon resonance [62]. We suggest this is understandable because the hydrodynamic force on the bead is too low to prevent thermal fluctuations [14], thus decreasing contact between surfaces. In addition, the specific engagement of ICAM-1 and anti-ICAM-1 should restrict the range of available molecular orientations, thus decreasing the probability of nonspecific interactions. ii) Our finding that nonspecific interactions were less sensitive to forces that specific ones might seem surprising. It must be argued that this is consistent with previous experimental studies made on protein-RNA interaction [63]. Note that wide differences were reported between nonspecific interactions detected between different surfaces [63]–[65], and certainly more work would be required to determine whether nonspecific interactions detected in our experiments displayed *bona fide* catch bond behavior.

Fourthly, it is important to exclude the possibility that reported bond strengthening might be an artefact due to progressive lengthening of the microsphere tether, thus decreasing the force on the bond. This hypothesis may be excluded as follows: the tether between the microsphere and the surface may be modeled as a freely jointed chain [66] consisting of approximately 4 links separated by flexible hinges. Since the rotation timescale of an immunoglobulin domain falls within the submicrosecond rate [67] and a force of more than 100 pN is required to unfold an immunoglobulin domain [68], no tether lengthening is expected in the timescale of bond strengthening we reported. Note also that this tether model may be used to support the hypothesis that the stress applied on bonds by the microsphere brownian motion is negligible as compared to the flow-generated forces. Indeed, it may be shown from standard statistical mechanics that the average force <F> exerted by a particle bound to a spring of stiffness s is (for one degree of freedom): <F> = (2sk_B_T/π)^1/2^, where k_B_ is Boltzmann’s constant and T is the absolute temperature. If we approximate the molecular link between a bead and a surface as a freely jointed chain with 4 segments of length a = 15 nm, the stiffness s is 3kT/4a^2^ [66], yielding an average force <F> of 0.19 pN, which is markedly lower than the hydrodynamic force that ranged between 1.6 and 4.8 pN in our experiments.

A fifth point is the accurate determination of single bond strengthening with a single measurable parameter. Indeed, while a number of results illustrated the multiplicity of binding states formed by ligand-receptor couples [14], [15], [27], [56], [57], there was a need for a simple way to deal with this complexity and provide a workable description of bond rupture. We think that the combination of parameters k(F,0) and a(F) meets with this requirement. It must be emphasized that a(F) should be considered as an empirical parameter and more work is required to relate it to the precise structure of interacting molecules.

A sixth point is that results obtained with ICAM-1 monomers were sufficiently accurate to allow us to predict the behavior of divalent ligands with a single fitted parameter (i.e. the rebinding rate k_r_). A fully quantitative fit was obtained for the lowest two forces, and a semi-quantitative fit for the highest force. It must be emphasized that these results might be deemed satisfactory, since we had to neglect the influence of the nanometer-scale topography of receptors and ligands on force sharing and rate of formation of a second bond when a particle was maintained at rest by a first bond.

A fairly unexpected finding was that the fitted value of the rebinding rate increased as a function of the applied force. While this might be due to a forced alignment of binding molecules and exclusion of a range of conformations incompatible with bond formation, a better definition of interacting surfaces would be required to discuss this point. Indeed, there is very little available information on the effect of forces on binding rates between surfaces coated with binding molecules (see also remarks in the methods section of [27]).

In conclusion, we provided a simple experimental and theoretical framework for comparing the behavior of monovalent and divalent attachments. In view of the known importance and wide occurrence of mutivalency, it would be instructive to apply this approach to a number of situations by varying the structure of surfaces, nature of ligand-receptor couples, and properties of connection between molecules and surfaces. This might provide a basis for a better understanding of the incompletely defined concept of avidity. Indeed, avidity is often used as a qualitative way of accounting for the efficiency of cell membrane receptors to bind to multivalent ligands, and it is felt to represent the capacity to form multivalent bonds. Avidity is thus different from affinity [69], which is a rigorously defined parameter accounting for the thermodynamics of a well-defined ligand-receptor couple. Avidity is closely related to the premium of multivalent over monovalent binding. Our results suggest that the bond strengthening rate parameter we defined accounts for part of avidity.
